# Effectiveness of an antimicrobial stewardship program using an automated antimicrobial surveillance system based on indication for antimicrobial administration

**DOI:** 10.1186/s40780-025-00528-0

**Published:** 2025-12-17

**Authors:** Mikiyasu Sakai, Takamasa Sakai, Toshitaka Watariguchi, Atsushi Kawabata, Mana Shirai, Mitsumi Kakimoto, Yuki Hirao, Fumiko Ohtsu

**Affiliations:** 1 Graduate School of Pharmacy, Meijo University, 150 Yagotoyama, Tempaku-ku, Nagoya, Aichi 468-8503 Japan; 2 Department of Pharmacy, Toyota Kosei Hospital, 500-1, Ibobara, Jousui-cho, Toyota, Aichi 470-0396 Japan; 3 Drug Informatics, Faculty of Pharmacy, Meijo University, 150 Yagotoyama, Tempaku-ku, Nagoya, Aichi 468-8503 Japan; 4 Department of General Internal Medicine, Toyota Kosei Hospital, 500-1, Ibobara, Jousui-cho, Toyota, Aichi 470-0396 Japan; 5 Department of Infectious Disease, Toyota Kosei Hospital, 500-1, Ibobara, Jousui-cho, Toyota, Aichi 470-0396 Japan; 6 Department of Pharmacy, Anjo Kosei Hospital, 28 Higashihirokute, Anjo-cho, Anjo, Aichi 446-8602 Japan; 7 Department of Pharmacy, Kainan Hospital, 396, Minamihonden, Maegasu-cho, Yatomi, Aichi 498-8502 Japan; 8 Department of Pharmacy, Konan Kosei Hospital, 137 Ohmatsubara, Takaya-cho, Konan, Aichi 483-8704 Japan

**Keywords:** Automated antimicrobial surveillance, Antimicrobial stewardship, Days of therapy

## Abstract

**Background:**

Conventional antimicrobial surveillance measures usage in days of therapy (DOT). However, assessments using DOT alone often ignore indication-specific details, which limits the identification of targets for antimicrobial stewardship (AS) interventions. Therefore, we have developed an automated surveillance system (the Antimicrobial and Patient Background Surveillance System [APBSS]) in order to analyze antimicrobial use based on indications, and evaluated the short-term effectiveness of an AS program using APBSS surveillance data, as an initial evaluation of the newly developed system.

**Methods:**

Using an interrupted time series design, we calculated the carbapenem DOT per 100 patient days as the primary outcome by comparing the pre-intervention (October 2022 to September 2024) and post-intervention (October 2024 to September 2025) periods. During the intervention, the AS team presented the APBSS surveillance data, including indication-specific antimicrobial use, patient characteristics, annual trends at our hospital, and multicenter comparisons. The antipseudomonal agent DOT was a secondary outcome.

**Results:**

Patient characteristics did not differ substantially between the periods. APBSS surveillance data identified key issues, including high carbapenem use in specific infections (e.g. Gram-negative rod bacteremia), which occurred despite the lower proportion of patients with hematological malignancies compared with other hospitals. However, the intervention significantly reduced carbapenem DOT levels (level change: −0.69/100 patient days, *p* = 0.017). The antipseudomonal agent DOT also significantly decreased (level change: −1.7/100 patient days, *p* = 0.004).

**Conclusions:**

The AS program that used APBSS surveillance data improved the use of carbapenems and antipseudomonal agents in the short-term. Thus, our initial evaluation has suggested that AS programs that use APBSS surveillance data can contribute to the appropriate use of antimicrobial agents.

**Supplementary Information:**

The online version contains supplementary material available at 10.1186/s40780-025-00528-0.

## Background

The number of deaths associated with bacterial antimicrobial resistance (AMR) was approximately 4.71 million in 2021 and is expected to rise to 8.22 million by 2050 [1]. A major contributing factor is an increase in carbapenem resistance. This necessitates the appropriate use of antibiotics, particularly carbapenems. In Japan, the 2023 National Action Plan on Antimicrobial Resistance aims to reduce the use of carbapenems by 20% compared to 2020 levels by 2027 [2]. The use of days of therapy (DOT), calculated as the number of days of antimicrobial use normalized to patient days, is recommended to survey trends in the use of antimicrobials, including carbapenems [3]. The Japan Surveillance for Infection Prevention and Healthcare Epidemiology (J-SIPHE) released an application that automatically calculates DOT. The J-SIPHE platform enables a comparison of DOT in facilities throughout Japan [4]. However, DOT comparisons alone have limitations in assessing appropriate use for specific diseases and identifying targets for intervention. Therefore, we developed an Antimicrobial and Patient Background Surveillance System (APBSS) that automatically calculates indication-based antimicrobial use and patient characteristics using the Diagnosis Procedure Combination (DPC) and Japan Nosocomial Infections Surveillance (JANIS) laboratory data. In our previous study, the APBSS identified indications for antimicrobial use (e.g., community-acquired infections, CAIs, healthcare-associated infections [HAIs], and surgical prophylaxis) with high accuracy using point prevalence surveys as reference data [5]. In addition, we identified the major CAIs with high accuracy. In other words, the APBSS has made it possible to visualize antimicrobial use trends and patient characteristics by specific infectious diseases, which could not be clarified by DOT alone. However, the effectiveness of an antimicrobial stewardship (AS) program using an APBSS has not been evaluated.

Toyota Kosei Hospital implemented the AS program in October 2020 (Table S1). However, this hospital tended to use carbapenems more frequently than other hospitals that claimed additional healthcare reimbursement for infection prevention and a control fee of 1 (Fig. S1) [6]. Therefore, we conducted an intervention within AS programs by presenting surveillance data created by APBSS (APBSS surveillance data).

In this study, we aimed to clarify the short-term effectiveness of the AS program using the APBSS by comparing the periods before and after its implementation, with the DOT of carbapenems as the primary outcome, as an initial evaluation of the newly developed system.

## Methods

### Study design

We conducted an interrupted time-series (ITS) study comparing the pre-intervention (October 2022–September 2024) and post-intervention (October 2024–September 2025) periods at Toyota Kosei Hospital. The ITS is a quasi-experimental design for evaluating the effects of an intervention by analyzing changes in the level and trends of outcomes before and after the intervention. Conventional before-and-after comparisons compare outcome statistics before and after the intervention. In contrast, the ITS increases validity in the estimation of intervention effects by considering temporal trends and autocorrelations and by assuming counterfactuals that would have continued the trend in the absence of the intervention [7].

### Patient characteristics

Patient characteristics were collected using DPC (EF files and Form 1) and JANIS laboratory data. The data collected included age, sex, length of hospital stay, hospital mortality, presence of solid tumors, hematological malignancy, Charlson Comorbidity Index, surgery during hospitalization, and bacteria detected in patients receiving antibiotics.

### AS program during study period

The AS program was conducted during the pre and post-intervention periods as listed in Table S1. All feedback to the prescribers was provided via entries in electronic health records. Prospective audits and feedback (PAF) for patients with bacteremia was performed when blood cultures turned positive and subsequently when species identification and susceptibility results became available. These patients were monitored until their condition stabilized. PAF for patients receiving anti-pseudomonal and anti-MRSA agents was performed when the targeted agent was initiated and when species identification and susceptibility results became available. Additionally, conferences with infectious disease specialists were held weekly and feedback was provided as needed. The monitoring of this group continued until the targeted agent was discontinued.

### Intervention

#### Preparation of APBSS surveillance data for the intervention

The DPC (EF files and Form 1) and JANIS laboratory data obtained between January 2019 and June 2024 were used to prepare the APBSS surveillance data at Toyota Kosei Hospital. For interfacility comparisons, DPC (EF files and Form 1) and JANIS laboratory data obtained between April 2022 and June 2024 were also used. In addition, data were collected from three other facilities with more than 500 beds belonging to the Aichi Prefectural Welfare Federation of Agricultural Cooperatives: Anjo Kosei Hospital, Konan Kosei Hospital, and Kainan Hospital. These data served as reference surveillance data for facilities with similar characteristics.

A brief data collection period after treatment initiation may have led to an underestimation of the treatment duration. For example, the treatment duration could be calculated as extremely short owing to the absence of subsequent follow-up data in cases where treatment was initiated in June 2024, the final month of the APBSS surveillance data collection period. To avoid underestimating the treatment duration owing to incomplete records, APBSS surveillance data creation was limited to cases in which the treatment initiation date was on or before March 31, 2024. This ensured a minimum follow-up period of three months for each case.

Surveillance data were curated for the following items to generate annual trend data at Toyota Kosei Hospital and multicenter comparison data: 1) proportion of indications (HAIs or CAIs) among patients using carbapenems, 2) DOT of antimicrobial agents by classes in patients with pneumonia, 3) DOT of antimicrobial agents by classes in patients with intra-abdominal infection, 4) DOT of antimicrobial agents by classes in patients with pyelonephritis, 5) DOT of antimicrobial agents by classes in patients with Gram-negative rod (GNR) bacteremia, 6) patient characteristics (age, sex, severity of pneumonia, length of therapy, length of stay, hospital mortality, solid tumors, hematological malignancy, Charlson Comorbidity Index, and detected bacteria) in patients with various infections. All surveillance data for three infections (pneumonia, intra-abdominal infections, and pyelonephritis) were calculated only for CAIs. The infections for which surveillance data were calculated were all confirmed to be identifiable with high accuracy using the APBSS [5]. For HAIs and CAIs other than these specific infections, surveillance data were not calculated because of the low accuracy in identifying specific infections for those categories. Carbapenems are commonly used to treat infections caused by GNR. Thus, surveillance data were obtained for patients with GNR bacteremia. The DOT (per case) was calculated as the number of days of antimicrobial use.

#### Intervention using APBSS surveillance data

APBSS surveillance data, as indicated in section “Preparation of APBSS surveillance data for the intervention” (items 1–6), were presented to the AS team on October 4, 2024, to discuss improvements in AS programs. These data were not disclosed to personnel outside the AS team.

#### AS program during the pre-intervention period

During the pre-intervention period, only DOT data were extracted quarterly from the J-SIPHE. In particular, as shown in Fig. S1, we used monthly DOT data from Toyota Kosei Hospital and other facilities in Japan that claimed additional healthcare reimbursement for infection prevention and control fees 1 and provided data to the J-SIPHE.

### Outcomes

The primary outcome was DOT for carbapenems. The secondary outcomes were DOT for antipseudomonal agents and DOT for carbapenems in patients with specific infections, including pneumonia, intra-abdominal infections, pyelonephritis, and GNR bacteremia. Additionally, data on DOT by antimicrobial class were extracted to examine changes in other antimicrobial classes. DOT data from the J-SIPHE were managed by the AMR Clinical Reference Center at the National Center for Global Health and Medicine. However, because the DOT for carbapenems in patients with specific infections could not be obtained from the J-SIPHE, these data were extracted using APBSS.

### Statistical analysis

All statistical analyses were performed using R software (version 4.3.3. (R Foundation for Statistical Computing, Vienna, Austria). An ITS segmented regression analysis was used to compare the pre- and post-intervention outcomes. Harmonic terms were added to the models to account for seasonality. Sensitivity analyses were also performed. We compared the distribution of DOT for carbapenems during the pre-intervention (October 2022 to September 2024) and post-intervention (October 2024 to September 2025) periods. We further compared the median values using the Mann-Whitney U test. Statistical significance was defined as *p* < 0.05. Additionally, carbapenem use is associated with hematological malignancy hospitalization [8–10]. Therefore, we calculated the DOT for carbapenems, excluding the antimicrobial agents used in patients admitted to the hematology department, and then conducted an ITS analysis. During the analysis of the primary outcome, we sought to confirm whether the DOT of carbapenems varied before and after the intervention owing to other factors, such as patient characteristics and drug shortages. Patient characteristics (age, sex, length of stay, etc.) were compared using the standardized mean difference (SMD). In addition, we sought to confirm the absence of a nationwide change due to other factors, such as the impact of drug shortages. We compared the median DOT of carbapenems among facilities that provided data to the J-SIPHE to claim additional healthcare reimbursement for infection prevention and control fees 1 by the ITS. Data from these facilities were extracted from the J-SIPHE on October 31, 2025.

## Results

### Patient characteristics

The patient characteristics are shown in Table 1. The SMDs between the pre- and post-intervention periods were all < 0.1, indicating no significant differences.

**Table 1:** Patient characteristics

Characteristic	**Pre-intervention period**, *n* = 31,986	**Post-intervention period**, *n* = 15,667	SMD
Age, years, mean (SD)	65 (22)	66 (21)	0.03
Sex, n (%)			−0.02
Female	14,321 (44.8%)	6,830 (43.6%)	
Male	17,665 (55.2%)	8,837 (56.4%)	
Length of stay, days, mean (SD)	12 (16)	12 (13)	−0.05
Hospital mortality, n (%)	1,744 (5.5%)	715 (4.6%)	−0.04
Solid tumor, n (%)	6,811 (21.3%)	3,392 (21.7%)	0.01
Hematological malignancy, n (%)	703 (2.2%)	414 (2.6%)	0.03
Charlson Comorbidity Index, mean (SD)	1.47 (2.00)	1.48 (1.98)	0.01
Surgery during hospitalization, n (%)	15,613 (48.8%)	7,726 (49.3%)	0.01
Pneumonia, n (%)	2,212 (6.9%)	1,204 (7.7%)	0.03
Intra-abdominal infection, n (%)	1,395 (4.4%)	650 (4.1%)	−0.01
Pyelonephritis, n (%)	827 (2.6%)	352 (2.2%)	−0.02
Gram-negative rod bacteremia, n (%)	788 (2.5%)	363 (2.3%)	−0.01
Detected bacteria, n (%) *			
Enterobacterales ECK	495 (1.5%)	239 (1.5%)	0.00
*Pseudomonas aeruginosa*	538 (1.7%)	241 (1.5%)	−0.01
3GC-nonS Enterobacterales (excluding ECK)	536 (1.7%)	256 (1.6%)	0.00

SMD: standardized mean difference. SD: standard deviation. ECK: *Enterobacter cloacae* complex, *Citrobacter freundii*, and *Klebsiella aerogenes.* 3GC-nonS: third-generation cephalosporin-nonsusceptible.  * Limited to patients receiving antibiotics

### Details of intervention

#### APBSS surveillance data used for the intervention

To identify the challenges with antimicrobial therapy at Toyota Kosei Hospital, we used the APBSS to visualize and evaluate data that could not be captured by conventional antimicrobial surveillance, including the classification of indications for specific antimicrobials, usage trend by infectious diseases, and patient characteristics.

Analysis of annual trends in our institution’s APBSS surveillance data revealed that the proportion of indications among patients receiving carbapenems used in CAIs remained stable at approximately 75% (Fig. S2). Regarding the DOT due to infectious diseases, the DOT for carbapenems increased, particularly for intra-abdominal infections, despite no substantial changes in patient characteristics. Furthermore, an increase in carbapenem DOT was also observed for GNR bacteremia, a condition targeted for auditing by the AS Team (Fig. S3, Tables S2–S5).

A multicenter comparison of the APBSS surveillance data showed no considerable differences in the proportion of indications among patients receiving carbapenems between facilities (Fig. S4). However, the DOTs and patient characteristics for various infections differed between the facilities (Fig. S5, Tables S6–9). This revealed that at Toyota Kosei Hospital, the carbapenem DOT for GNR bacteremia was high despite the lower proportion of patients with hematological malignancies.

#### Challenges and countermeasures for AS at Toyota Kosei Hospital, as derived from APBSS surveillance data

Analysis of the APBSS surveillance data identified the appropriate use of carbapenems for intra-abdominal infections and GNR bacteremia as a key challenge. The AS team deliberated on these findings and established the following action plan: 1) The use of fourth-generation cephalosporins, fluoroquinolones, or trimethoprim-sulfamethoxazole was considered for infections caused by Enterobacterales at moderate risk of clinically significant inducible AmpC production, given the effectiveness of these agents [11]. 2) The use of carbapenems to target *Pseudomonas aeruginosa* was avoided (except in cases where resistance to other β-lactams had been acquired). This countermeasure was based on reports that carbapenems can readily induce resistance and on recommendations to select noncarbapenem β-lactam agents to which the organism is susceptible [11–14]. 3) The use of cefmetazole was considered for treating infections such as pyelonephritis and intra-abdominal infections, caused by extended-spectrum β-lactamase-producing organisms, in light of reports demonstrating its effectiveness [15, 16].

### Outcome

#### Changes in the overall DOT

**Fig. 1:**
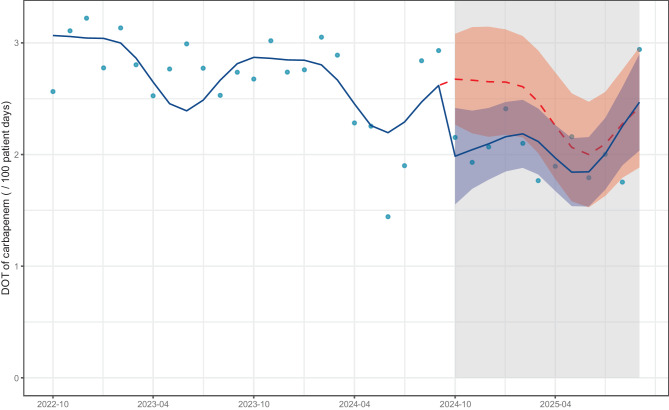
Interrupted time series analysis of dot for carbapenem in the pre- and post-intervention periods. The blue dots represent the observed values. The solid blue line represents the predicted trends. The dashed red line indicates the counterfactual scenario. The shaded areas indicate 95% confidence intervals for the observed trend (blue) and counterfactual scenario (red). dot: days of therapy

**Fig. 2:**
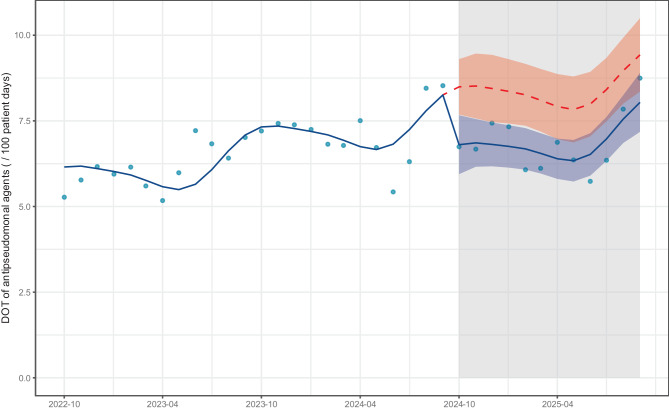
Interrupted time series analysis of dot for antipseudomonal agents in the pre- and post-intervention periods. The blue dots represent the observed values. The solid blue line represents the predicted trends. The dashed red line indicates the counterfactual scenario. The shaded areas indicate 95% confidence intervals for the observed trend (blue) and counterfactual scenario (red). dot: days of therapy

**Fig. 3:**
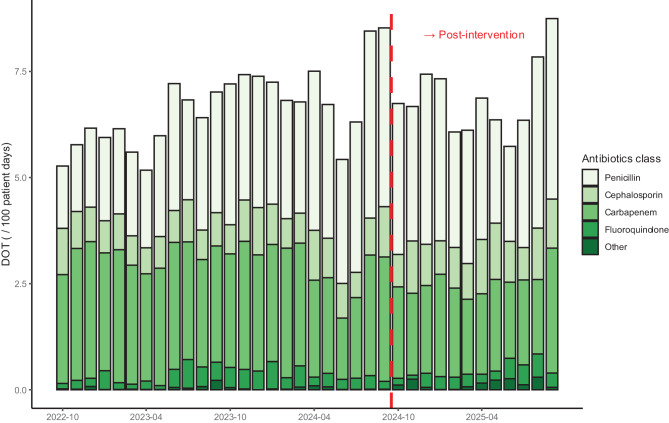
Trends in the DOTs of antipseudomonal agents, as distinguished by class. dot; days of therapy

The DOT (/100 patient days) for carbapenems was significantly reduced in the post-intervention period (level change: −0.69, 95% confidence interval [CI]: −1.2 to −0.13, *p* = 0.017), a significant, slightly positive slope change was also observed (slope change: 0.07, 95% CI: 0.00 to 0.13, *p* = 0.045) (Fig. 1, Table S10). The DOTs of all classes of antimicrobial agents are shown in Fig. S6. Sensitivity analysis comparing the outcomes showed a significant decrease in DOT (/100 patient days) for carbapenems in the post-intervention period (median [interquartile range (IQR)]: 2.77 [2.56, 2.95] vs. 2.04 [1.87, 2.15], *p* < 0.001; Fig. S7). Additionally, in the sensitivity analysis excluding antimicrobial agents used for patients admitted to the hematology department, carbapenem DOT (/100 patient days) significantly decreased in the post-intervention period (level change: −0.60, 95% CI: −1.1 to −0.15, *p* = 0.011), and no significant slope change was observed (slope change: 0.04, 95% CI: −0.02 to 0.09, *p* = 0.20) (Fig. S8, Table S11). The DOT (/100 patient days) for antipseudomonal agents, the secondary outcome, was significantly reduced in the post-intervention period (level change: −1.7, 95% CI: −2.8 to −0.58, *p* = 0.004), and no significant slope change was observed (slope change: 0.03, 95% CI: −0.10 to 0.16, *p* = 0.70) (Fig. 2, Table S12). The DOTs of antipseudomonal agents by class are shown in Fig. 3.

The DOT (/100 patient days) for carbapenems in facilities claiming additional healthcare reimbursement for infection prevention and control fee 1 showed no change during the post-intervention period (level change: 0.08, 95% CI: −0.03, 0.19, *p* = 0.13) (slope change: 0.00, 95% CI: −0.02 to 0.01, *p* = 0.70) (Fig. S9, Table S13). Notably, as of October 31, 2025, 1,356 facilities provided information on antimicrobial use for hospitalization. Of these, 969 (71.5%) uploaded data for September 2025, the last month of the analysis period.

#### Changes in the DOT with specific infectious diseases

**Fig. 4:**
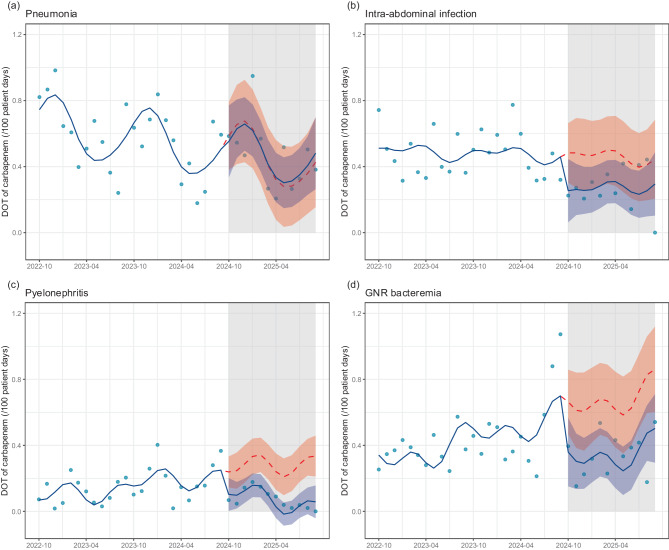
Interrupted time series analysis of carbapenem dot for four infections. This figure presents interrupted time series analyses of carbapenem dot for (**a**) pneumonia, (**b**) intra-abdominal infection, (**c**) pyelonephritis, and (**d**) Gram-negative rod (GNR) bacteremia, comparing pre- and post-intervention periods. The blue dots represent the observed values. The solid blue line represents the predicted trends. The dashed red line indicates the counterfactual scenario. The shaded areas indicate 95% confidence intervals for the predicted trend (blue) and counterfactual scenario (red). DOT: days of therapy

Figure 4 and Table S14 shows the results of the secondary outcome: trends in the DOT of carbapenems in the patients with specific infectious diseases. For patients with pneumonia, no significant change in DOT was observed after the intervention (level change: −0.03, 95% CI: −0.31 to 0.25, *p* = 0.80). In contrast, the DOT for carbapenems in patients with intra-abdominal infections reduced, although the difference was not statistically significant (level change: −0.23, 95% CI: −0.48 to 0.02, *p* = 0.068). Furthermore, the DOT for carbapenems was significantly reduced in patients with pyelonephritis (level change: −0.14, 95% CI: −0.26, −0.01, *p* = 0.038) and GNR bacteremia (level change: −0.30, 95% CI: −0.57, −0.04, *p* = 0.027). Moreover, no significant slope changes were observed for any of the infections. The DOT by antimicrobial class for intra-abdominal infections and GNR bacteremia identified as challenges for the AS program is shown in Fig. S10. For GNR bacteremia, the DOT of cephamycins and oxacephems increased post-intervention.

## Discussion

In this study, as an initial evaluation of the newly developed system, we have evaluated the effectiveness of AS programs using the APBSS surveillance data over a one-year period following intervention. To this end, we set the intervention point in the ITS as the time of presentation of APBSS surveillance data to the AS team. Additionally, we used the DOT as the outcome, which is recommended as an indicator for an AS program [3]. This intervention significantly decreased the DOT for carbapenems, whereas no change was observed in the nationwide surveillance data from facilities with claims of additional healthcare reimbursement for infection prevention and control fee 1. This discrepancy between our institution data and nationwide surveillance data emphasizes that the observed effect was due to the intervention, as other factors such as carbapenem drug shortages did not influence the primary outcome during the study period. Our results suggest that the APBSS surveillance data can contribute to the identification of AS problems and improve AS programs.

In AS programs, PAF are recommended for patients with bacteremia and broad-spectrum antimicrobial use. In addition, interventions for common infections, such as community-acquired pneumonia and urinary tract infections, are considered important [3, 17]. AS programs for specific diseases may effectively reduce the use of antipseudomonal agents and carbapenems [18]. However, the human resources for AS programs are limited in Japan [19]. A nationwide survey of AS programs collected data only on PAF in patients treated with specific antimicrobial agents; no data were collected on the status of AS programs for specific diseases [20]. Although AS programs for patients with bacteremia have been reported in Japan [21–23], those for other diseases are not as common. The APBSS provides representative disease-specific surveillance data, including those for diseases typically not targeted by AS programs in Japan. Furthermore, the efficiency of the APBSS is well established, imposing a low burden on those conducting surveillance [5]. Because of these features, we were able to effectively identify challenges in our hospital’s antimicrobial treatment, and interventions were implemented to address these challenges. Consequently, as shown in Fig. 4, we noted a significant decrease in the DOT of carbapenems for GNR bacteremia which had previously shown an upward trend at our hospital. In intra-abdominal infections, the DOT of carbapenems decreased, although the difference was not statistically significant. Conversely, no fluctuations were observed in the carbapenem DOT for pneumonia, an area not flagged as a concern. These findings indicate that interventions utilizing APBSS surveillance data are instrumental in reducing the use of broad-spectrum antimicrobials. However, a significant decrease in the DOT of carbapenems was observed in patients with pyelonephritis, which had not been identified as a challenge in the AS program at our hospital. This is likely because cases of pyelonephritis were included in those with GNR bacteremia, and this result is considered to be an associated effect of reduced carbapenem use for GNR bacteremia.

Carbapenem use is associated with hospitalization for hematological malignancy [8–10]. Nevertheless, APBSS surveillance data revealed that Toyota Kosei Hospital had a carbapenem DOT comparable to or even higher than that of other facilities, despite the lower proportion of patients with hematological malignancies among those with GNR bacteremia. We investigated whether this trend could be attributed to the detected bacteria but found no considerable differences in the distribution of bacterial isolates between the facilities. Specifically, the proportions of 1) Enterobacterales at moderate risk for clinically significant inducible AmpC production (*Enterobacter cloacae* complex, *Klebsiella aerogenes*, and *Citrobacter freundii*); 2) *P. aeruginosa*; and 3) third-generation cephalosporin-resistant Enterobacterales (excluding the former group) did not differ among the facilities. These findings suggest that the high carbapenem DOT at the Toyota Kosei Hospital cannot be explained by patient characteristics or pathogen profiles. Instead, we hypothesized that carbapenem-sparing therapy would not be effectively implemented through PAF of the AS team. To address this issue, countermeasures developed by the AS team fostered enhanced awareness and practice of carbapenem-sparing therapy, which subsequently reduced the carbapenem DOT for GNR bacteremia. This case demonstrates that creating antimicrobial surveillance data enriched with patient characteristics and indications for antimicrobial use enables comprehensive identification of challenges, thereby facilitating targeted improvements in the AS program.

This study has several limitations. First, we may not have completely eliminated confounders that affected the primary outcome. This is of particular concern, as our primary outcome, the DOT of carbapenems, showed considerable variation. To mitigate this limitation, we confirmed that there was no substantial variation in patient characteristics between the pre- and post-intervention periods. We also compared carbapenem DOT in the pre- and post-intervention periods using data from across Japan to confirm the absence of a nationwide change from other factors and found no variation. Second, the outcomes were evaluated at a single center. Therefore, it is necessary to verify whether similar results can be obtained at other facilities. Third, the post-intervention observation period was only 12 months, which prevented the verification of the long-term effects. In the main analysis, a notable increase in carbapenem DOT was observed in September 2025, the final month of the post-intervention period, resulting in a slightly positive slope change. This observation suggests that the initial level of change induced by the intervention might diminish on the long term. In contrast, in the sensitivity analysis excluding antimicrobial agents used for patients admitted to the hematology department, the carbapenem DOT in September 2025 was comparable to that immediately after the intervention (October 2024), and no slope change was observed. This suggests that it is highly likely that the increase in carbapenem DOT observed in the final month post-intervention was largely attributable to an increase in its use among patients with hematological malignancies, which is a factor unrelated to the intervention itself. However, this finding raises the possibility that the intervention effects were not sustained. The intervention was conducted only once in October 2024, and its long-term effectiveness needs to be verified in future studies.

## Conclusions

In this study, intervention through an AS program using APBSS surveillance data improved the use of carbapenems and antipseudomonal agents in the short-term. Thus, our initial evaluation suggests that AS programs that use APBSS surveillance data can contribute to the appropriate use of antimicrobial agents.

## Electronic supplementary material

Below is the link to the electronic supplementary material.

Supplementary Material 1

## Data Availability

The datasets used and/or analyzed in the current study are available from the corresponding author upon reasonable request.

## Abbreviations

AMR: antimicrobial resistance
DOT: days of therapy
J-SIPHE: Japan Surveillance for Infection Prevention and Healthcare Epidemiology
APBSS: Antimicrobial and Patient Background Surveillance System
DPC: Diagnosis Procedure Combination; JANIS, Japan Nosocomial Infections Surveillance
CAIs: Community-acquired infections
HAIs: Healthcare-associated infections
AS: Antimicrobial stewardship
ITS: Interrupted time series
GNR: Gram-negative rod
SMD: Standardized mean difference
CI: Confidence interval
IQR: Interquartile range
PAF: Prospective audit and feedback
