# Supplementary material for: Effectiveness of an antimicrobial stewardship program using an automated antimicrobial surveillance system based on indication for antimicrobial administration
Source: J Pharm Health Care Sci. 2025 Dec 17;12:9. doi: 10.1186/s40780-025-00528-0 (PMC12821170; doi:10.1186/s40780-025-00528-0)
Supplement: Supplementary file 1 — Supplementary Material 1 [file 40780_2025_528_MOESM1_ESM.docx]

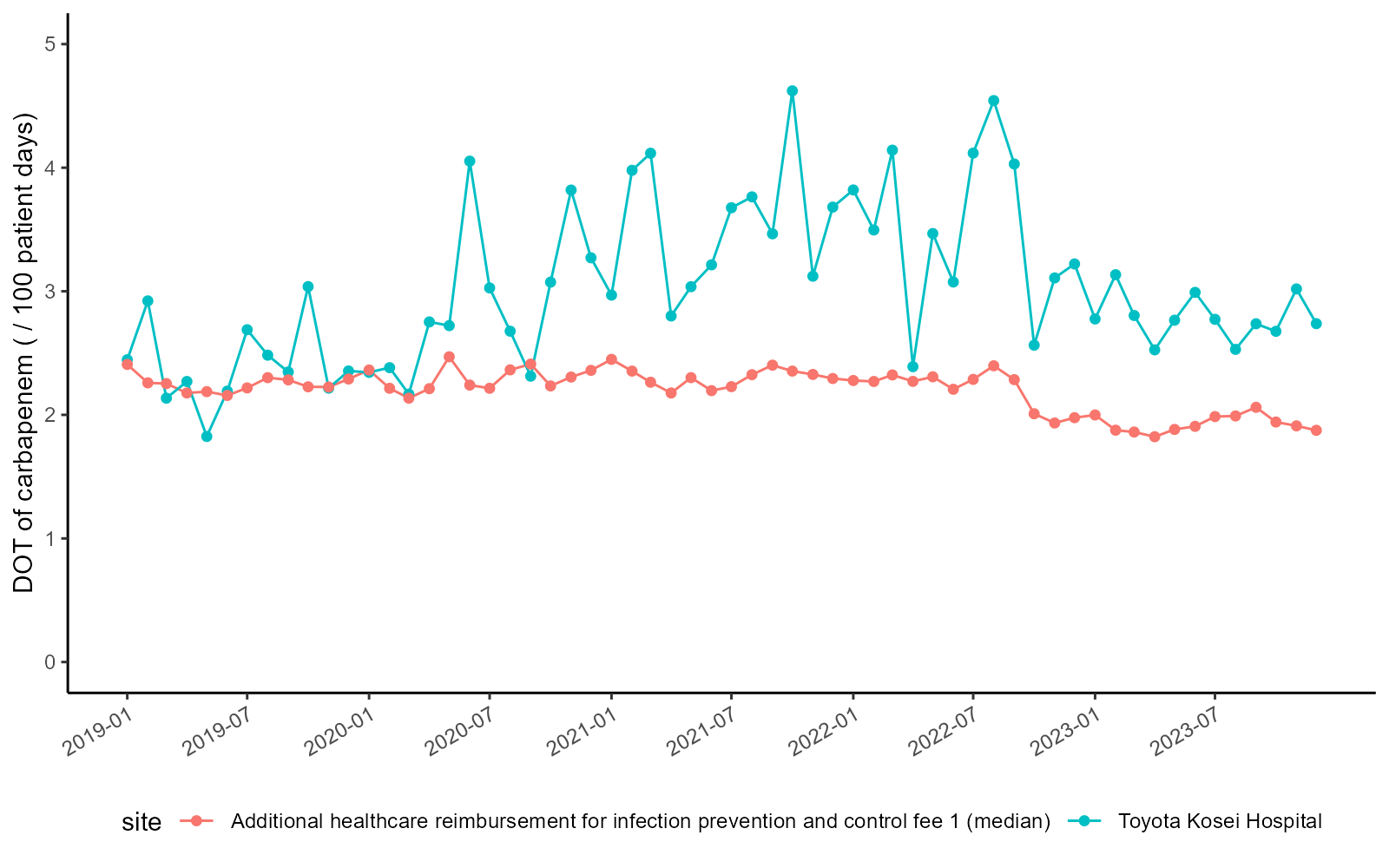


Fig. S1. Days of therapy (DOT) with carbapenems at Toyota Kosei Hospital and at hospitals claiming additional healthcare reimbursement for infection prevention and control fee 1.


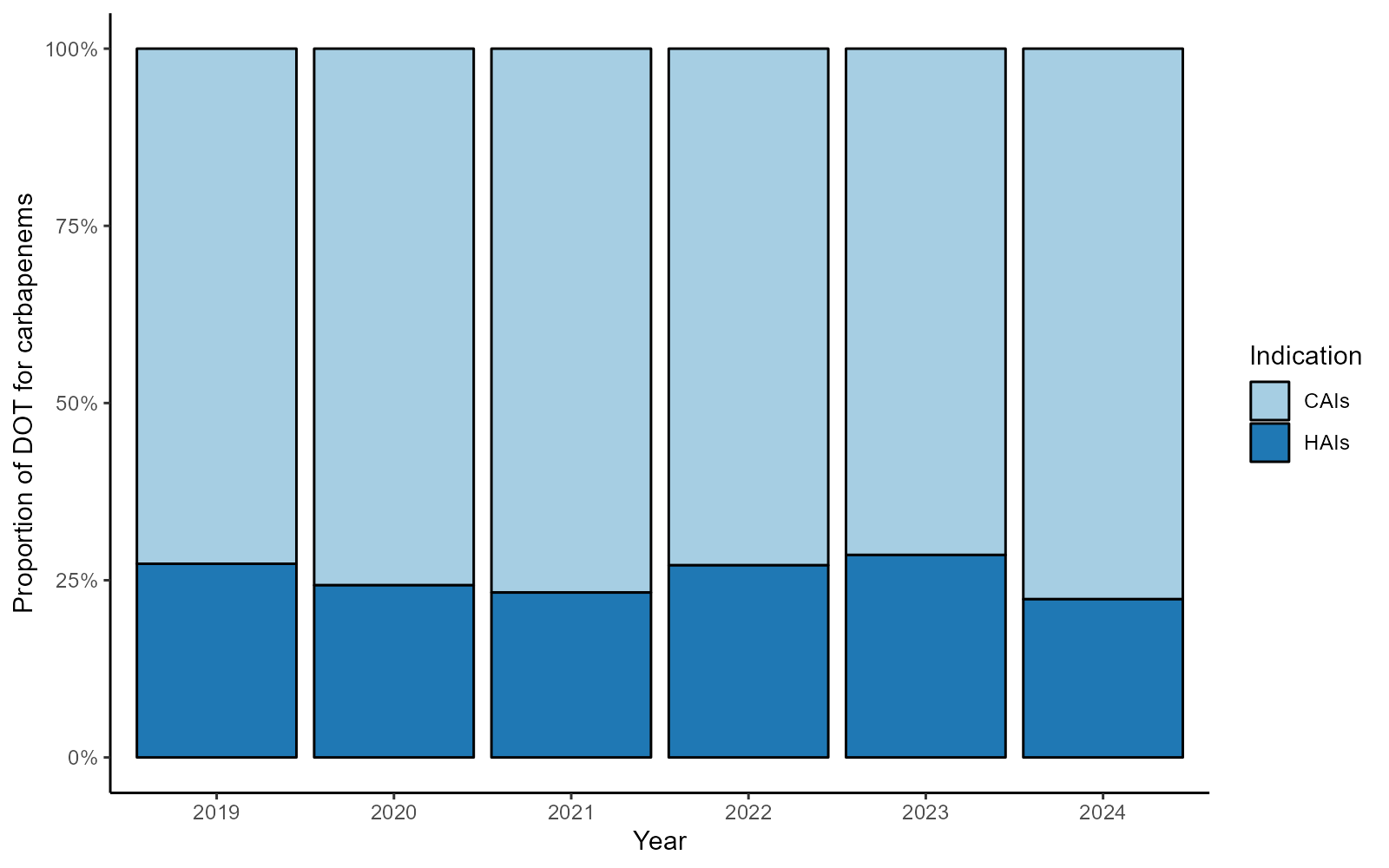


Fig. S2. Annual trends in carbapenem indications at Toyota Kosei Hospital.

Data were generated via the Antimicrobial and Patient Background Surveillance System (APBSS) using cases in which antimicrobial treatment was initiated by March 2024.

DOT; days of therapy

CAIs: community-acquired infections

HAIs: healthcare-associated infections.


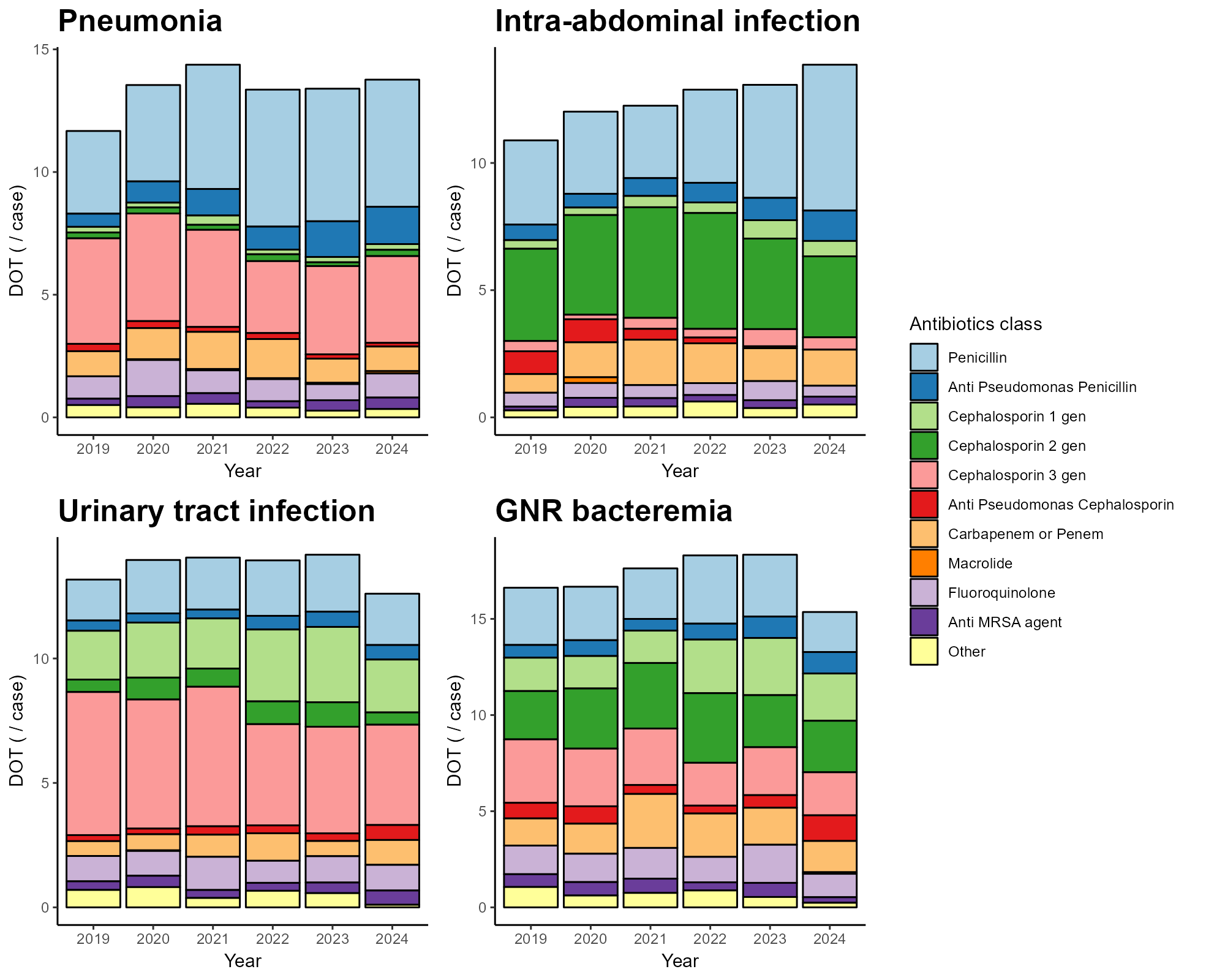


Fig. S3. Annual trends in DOT for various infectious diseases at Toyota Kosei Hospital.

Data were generated via the Antimicrobial and Patient Background Surveillance System (APBSS) using cases in which antimicrobial treatment was initiated by March 2024.

DOT; days of therapy

GNR; gram negative rod

MRSA; methicillin-resistant *Staphylococcus aureus*.
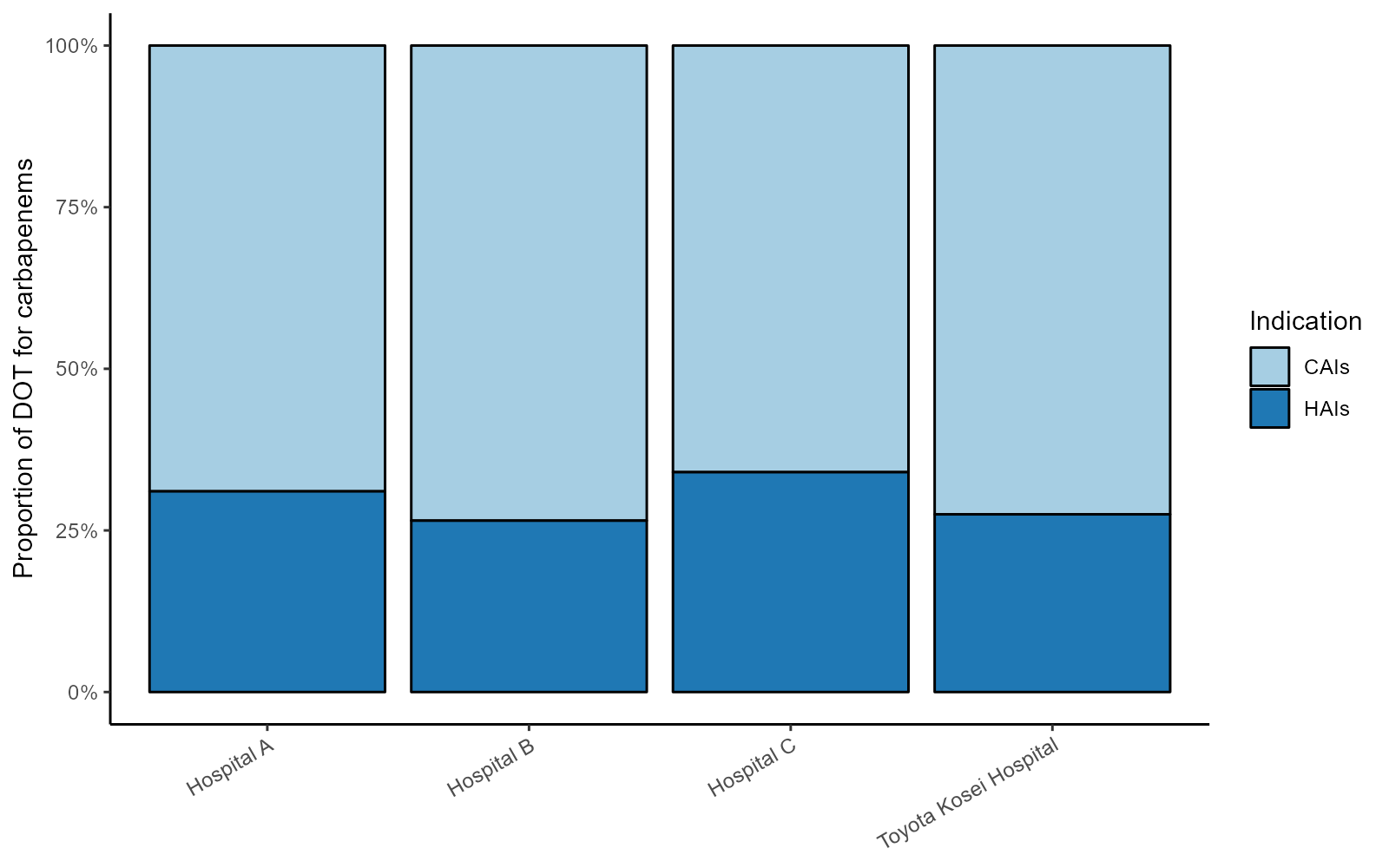


Fig. S4. Indications for carbapenems in various hospitals.

Data were generated via the Antimicrobial and Patient Background Surveillance System (APBSS) using cases in which antimicrobial treatment was initiated by March 2024.

DOT; days of therapy

CAIs: community-acquired infections

HAIs: healthcare-associated infections.


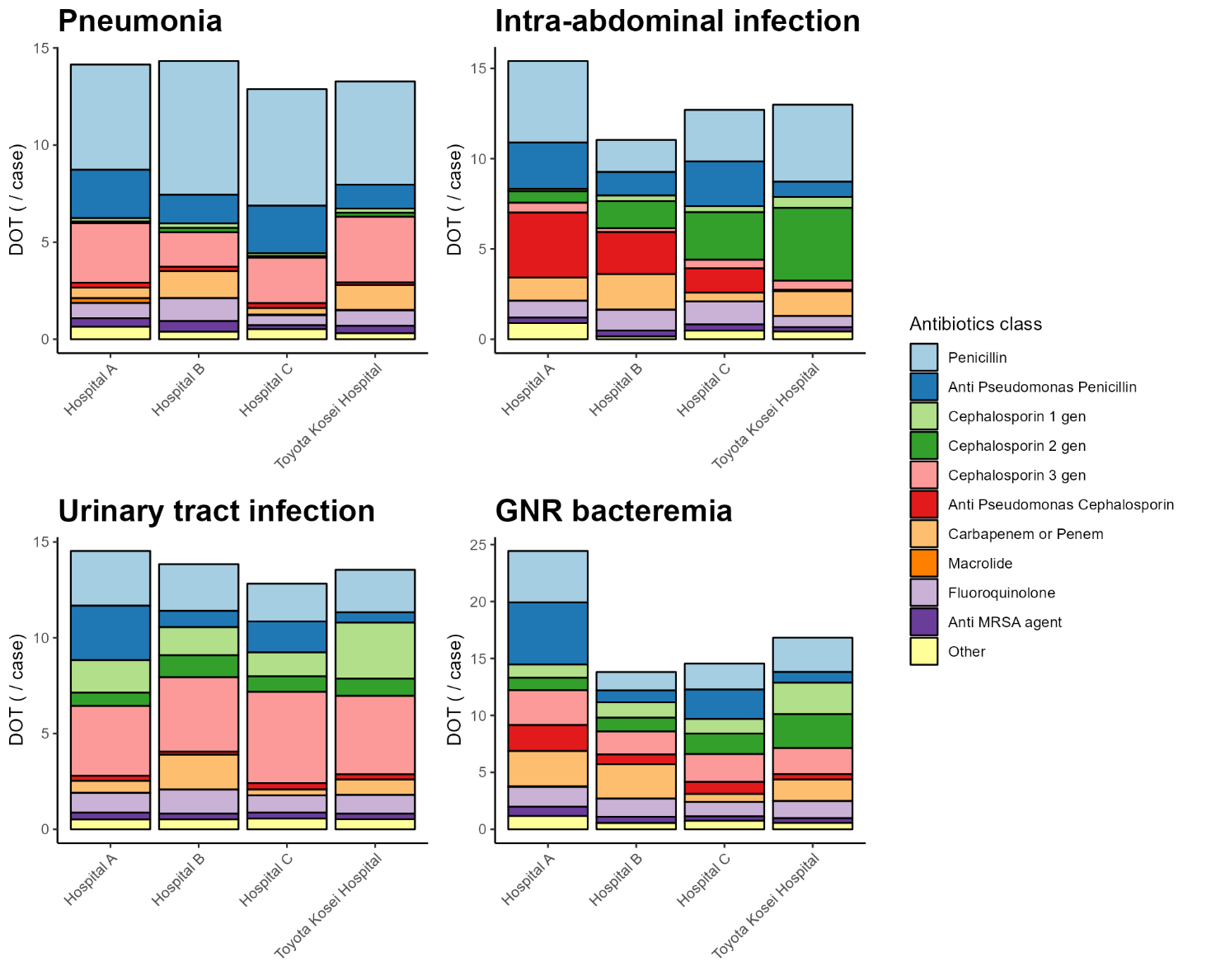


Fig. S5. DOT for various infectious diseases in various hospitals.

Data were generated via the Antimicrobial and Patient Background Surveillance System (APBSS) using cases in which antimicrobial treatment was initiated by March 2024.

GNR; gram negative rod

MRSA; methicillin-resistant *Staphylococcus aureus*.


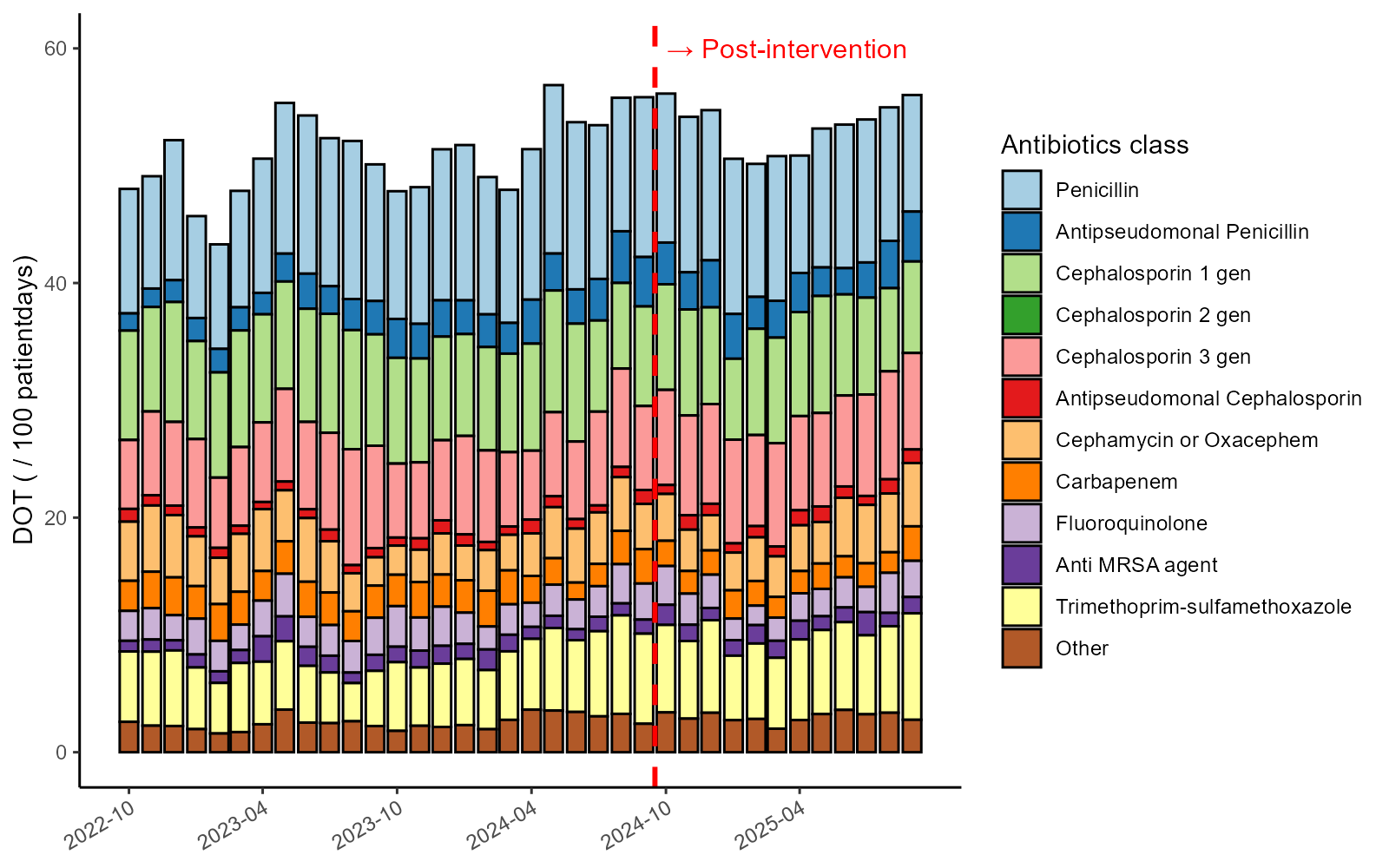


Fig. S6. Trends in the DOTs, as distinguished by class.

DOT; days of therapy.

MRSA; methicillin-resistant *Staphylococcus aureus*.


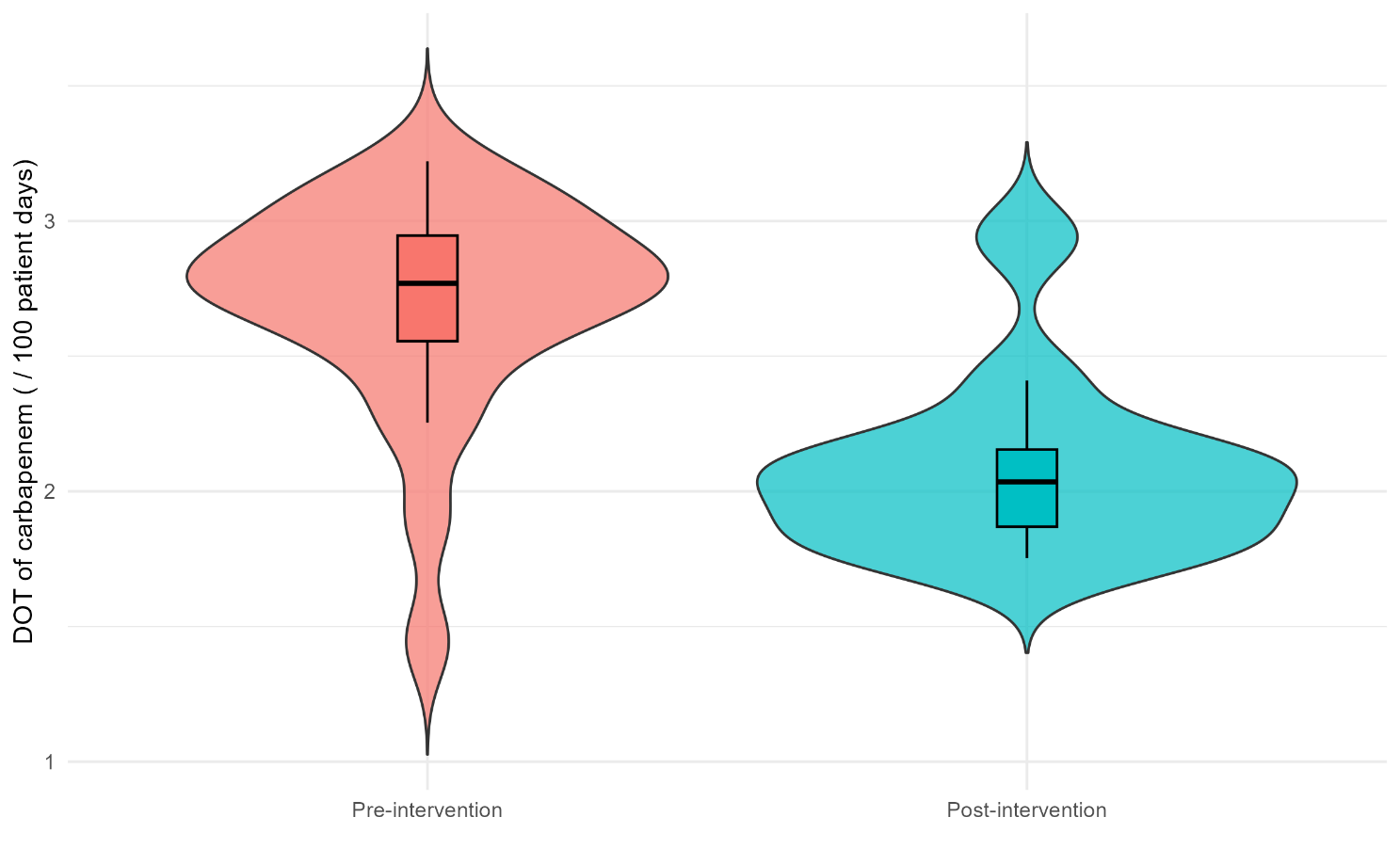


Fig. S7. Comparison of DOT for carbapenem pre- and post-intervention.

DOT; days of therapy.


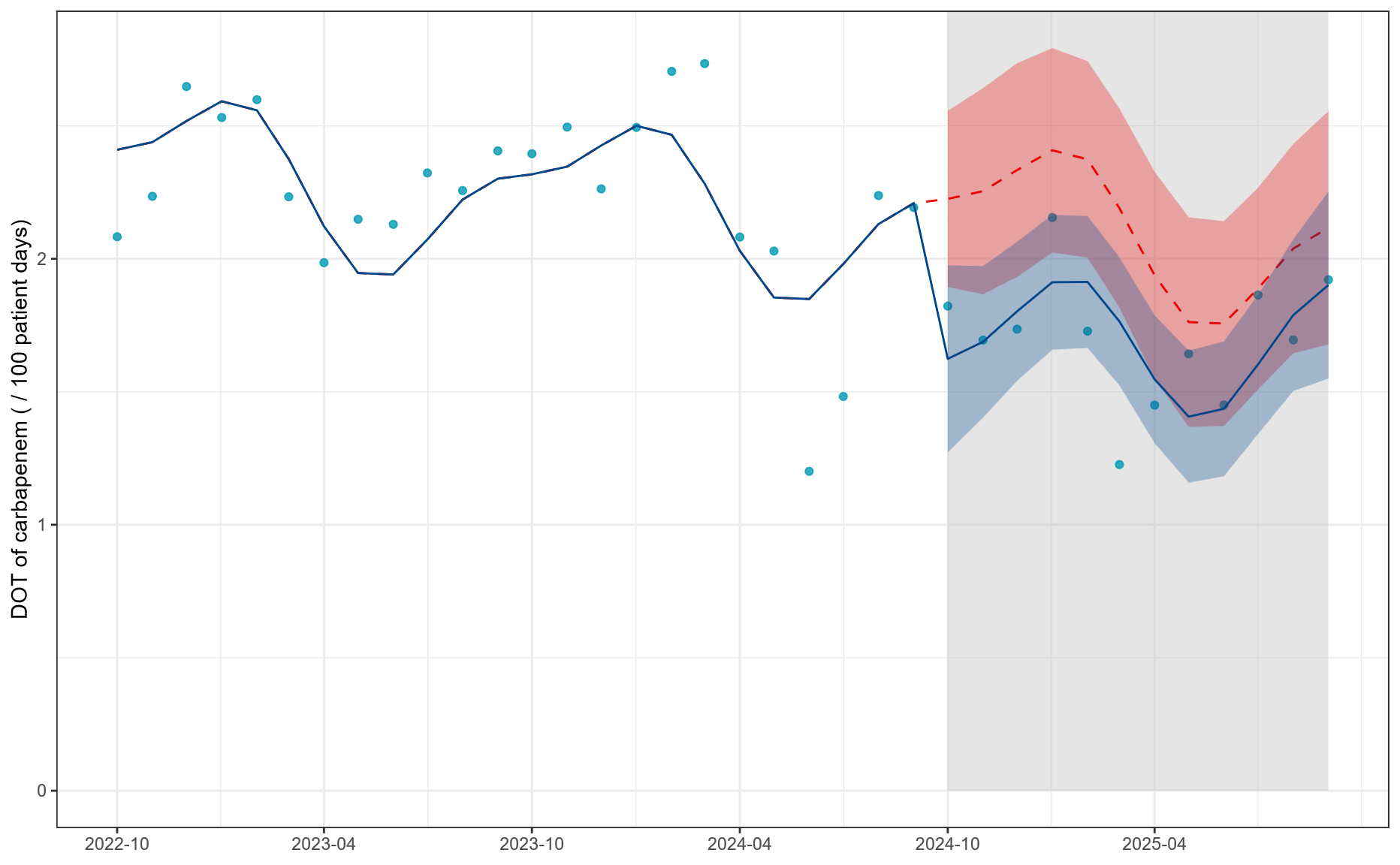


Fig. S8. Interrupted time series analysis of DOT for carbapenem excluding use in the hematology department, in the pre- and post-intervention periods

The blue dots represent the observed values. The solid blue line represents the predicted trends. The dashed red line indicates the counterfactual scenario. The shaded areas indicate 95% confidence intervals for the observed trend (blue) and counterfactual scenario (red). DOT: days of therapy.


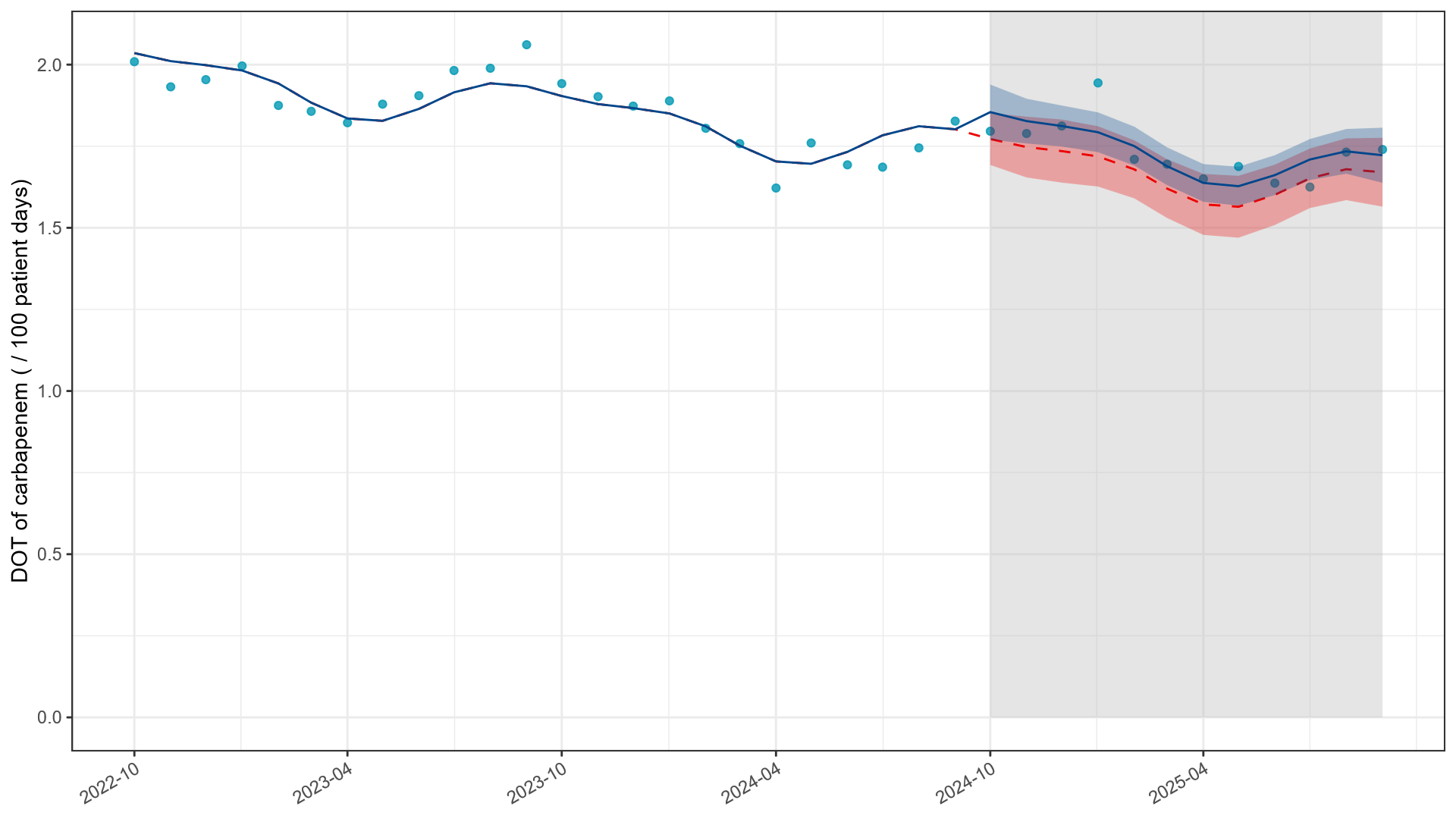


Fig. S9. Interrupted time-series analysis of carbapenem for DOT in hospitals with additional healthcare reimbursement for infection prevention and control fee 1.

The blue dots represent the observed values. The solid blue line represents the predicted trends. The dashed red line indicates the counterfactual scenario. The shaded areas indicate 95% confidence intervals for the observed trend (blue) and counterfactual scenario (red). DOT: days of therapy.


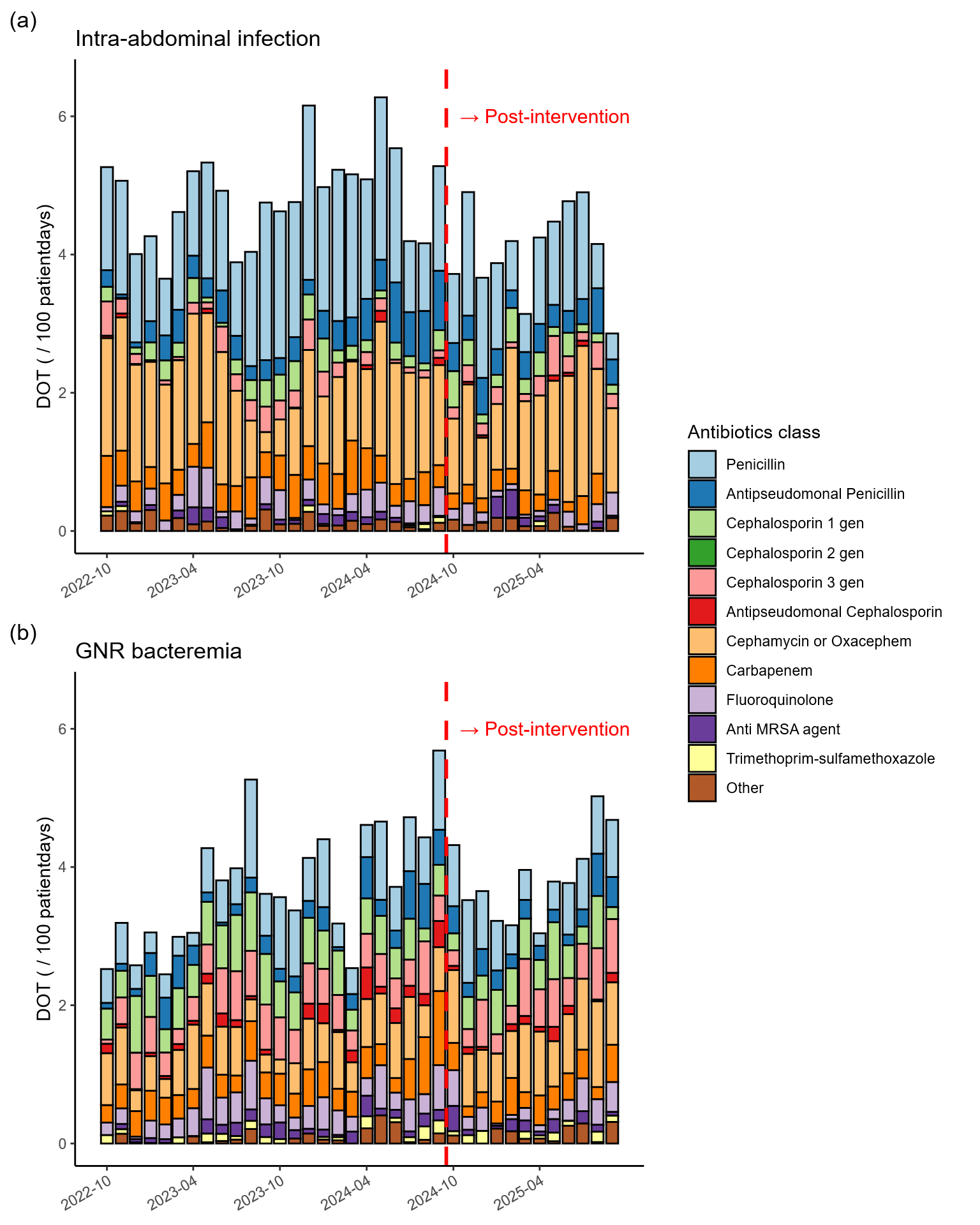


Fig. S10. Trends in the DOTs, as distinguished by class in two infections identified as key challenge for an antimicrobial stewardship in Toyota Kosei hospital.

The stacked bar charts display the monthly antibiotic DOT per 100 patient days, stratified by antibiotic class, for (a) Intra-abdominal infection and (b) GNR bacteremia. DOT: days of therapy, GNR: gram negative rod, MRSA: methicillin-resistant *Staphylococcus aureus*.

Table S1. Antimicrobial stewardship program at Toyota Kosei Hospital

| Member | Interventions |
| --- | --- |
| Infectious disease physician | PAF for patients with bacteremia |
| General medicine physician | PAF for patients with bacteremia in the absence of an infectious disease physician |
| Pharmacist | PAF for patients administered anti-pseudomonal, anti-MRSA agents |

PAF: prospective audit and feedback

MRSA; methicillin-resistant *Staphylococcus aureus*.

Table S2. Annual trends in the characteristics of patients with pneumonia at Toyota Kosei Hospital

| **Characteristics** | **2019**,  N = 868^1^ | **2020**,  N = 867^1^ | **2021**,  N = 868^1^ | **2022**,  N = 923^1^ | **2023**,  N = 1,100^1^ | **2024**,  N = 285^1^ |
| --- | --- | --- | --- | --- | --- | --- |
| Age, years | 78 (66, 86) | 81 (72, 88) | 81 (72, 87) | 82 (74, 89) | 81 (68, 87) | 82 (74, 88) |
| Sex |  |  |  |  |  |  |
| Female | 329 (37.9%) | 314 (36.2%) | 312 (35.9%) | 351 (38.0%) | 434 (39.5%) | 110 (38.6%) |
| Male | 539 (62.1%) | 553 (63.8%) | 556 (64.1%) | 572 (62.0%) | 666 (60.5%) | 175 (61.4%) |
| ADROP score |  |  |  |  |  |  |
| 0 | 54 (6.2%) | 56 (6.5%) | 26 (3.0%) | 17 (1.8%) | 22 (2.0%) | 4 (1.4%) |
| 1 or 2 | 297 (34.2%) | 303 (34.9%) | 142 (16.4%) | 148 (16.0%) | 257 (23.4%) | 58 (20.4%) |
| 3 | 118 (13.6%) | 126 (14.5%) | 64 (7.4%) | 49 (5.3%) | 110 (10.0%) | 39 (13.7%) |
| 4 or 5 | 40 (4.6%) | 60 (6.9%) | 31 (3.6%) | 20 (2.2%) | 41 (3.7%) | 8 (2.8%) |
| Unknown | 359 (41.4%) | 322 (37.1%) | 605 (69.7%) | 689 (74.6%) | 670 (60.9%) | 176 (61.8%) |
| Length of therapy, days | 9 (6, 13) | 10 (7, 14) | 10 (7, 15) | 10 (7, 14) | 9 (6, 14) | 10 (7, 14) |
| Length of stay, days | 14 (7, 25) | 17 (9, 30) | 18 (10, 31) | 19 (10, 33) | 16 (8, 31) | 18 (11, 34) |
| Hospital mortality | 141 (16.3%) | 130 (15.1%) | 160 (18.6%) | 204 (22.6%) | 206 (19.0%) | 60 (21.4%) |
| Solid tumor | 154 (17.7%) | 165 (19.0%) | 147 (16.9%) | 127 (13.8%) | 161 (14.6%) | 50 (17.5%) |
| Hematological malignancy | 23 (2.6%) | 26 (3.0%) | 14 (1.6%) | 28 (3.0%) | 31 (2.8%) | 4 (1.4%) |
| Charlson Comorbidity Index | 2 (0, 3) | 2 (0, 3) | 2 (0, 2) | 2 (0, 2) | 2 (0, 2) | 2 (0, 3) |
| Detected bacteria |  |  |  |  |  |  |
| *S. pneumoniae* | 44 (5.1%) | 33 (3.8%) | 25 (2.9%) | 20 (2.2%) | 36 (3.3%) | 8 (2.8%) |
| *M. catarrhalis* | 31 (3.6%) | 16 (1.8%) | 17 (2.0%) | 16 (1.7%) | 33 (3.0%) | 10 (3.5%) |
| *H. influenzae* | 52 (6.0%) | 39 (4.5%) | 15 (1.7%) | 24 (2.6%) | 69 (6.3%) | 19 (6.7%) |
| Enterobacterales ECK | 37 (4.3%) | 36 (4.2%) | 54 (6.2%) | 54 (5.9%) | 58 (5.3%) | 12 (4.2%) |
| *P. aeruginosa* | 57 (6.6%) | 61 (7.0%) | 66 (7.6%) | 68 (7.4%) | 68 (6.2%) | 22 (7.7%) |
| *Acinetobacter* spp. | 12 (1.4%) | 12 (1.4%) | 14 (1.6%) | 15 (1.6%) | 17 (1.5%) | 2 (0.7%) |
| 3GC-nonS Enterobacterales (excluding ECK) | 24 (2.8%) | 28 (3.2%) | 33 (3.8%) | 25 (2.7%) | 28 (2.5%) | 12 (4.2%) |
| ^1^Median (IQR); n (%) | | | | | | |

ECK: *Enterobacter cloacae* complex, *Citrobacter freundii*, *Klebsiella aerogenes*

3GC-nonS, third-generation cephalosporin-non-susceptible

Data were generated via the Antimicrobial and Patient Background Surveillance System (APBSS) using cases in which antimicrobial treatment was initiated by March 2024.

Table S3. Annual trends in the characteristics of patients with intra-abdominal infections at Toyota Kosei Hospital

| **Characteristics** | **2019**,  N = 520^1^ | **2020**,  N = 682^1^ | **2021**,  N = 775^1^ | **2022**,  N = 715^1^ | **2023**,  N = 702^1^ | **2024**,  N = 165^1^ |
| --- | --- | --- | --- | --- | --- | --- |
| Age, years | 71 (54, 80) | 73 (58, 81) | 74 (57, 83) | 74 (59, 84) | 74 (59, 84) | 75 (63, 83) |
| Sex |  |  |  |  |  |  |
| Female | 209 (40.2%) | 275 (40.3%) | 318 (41.0%) | 285 (39.9%) | 300 (42.7%) | 74 (44.8%) |
| Male | 311 (59.8%) | 407 (59.7%) | 457 (59.0%) | 430 (60.1%) | 402 (57.3%) | 91 (55.2%) |
| Length of therapy, days | 9 (7, 13) | 10 (7, 14) | 9 (6, 14) | 10 (7, 14) | 9 (6, 13) | 10 (7, 14) |
| Length of stay, days | 10 (7, 18) | 10 (7, 18) | 11 (7, 19) | 12 (8, 18) | 11 (8, 19) | 13 (9, 19) |
| Hospital mortality | 21 (4.1%) | 28 (4.1%) | 38 (4.9%) | 39 (5.5%) | 31 (4.5%) | 14 (8.6%) |
| Solid tumor | 122 (23.5%) | 174 (25.5%) | 180 (23.2%) | 167 (23.4%) | 172 (24.5%) | 37 (22.4%) |
| Hematological malignancy | 2 (0.4%) | 7 (1.0%) | 7 (0.9%) | 3 (0.4%) | 7 (1.0%) | 0 (0.0%) |
| Charlson Comorbidity Index | 0 (0, 2) | 1 (0, 2) | 0 (0, 2) | 1 (0, 2) | 1 (0, 2) | 0 (0, 2) |
| Detected bacteria |  |  |  |  |  |  |
| Enterobacterales ECK | 20 (3.8%) | 32 (4.7%) | 50 (6.5%) | 37 (5.2%) | 40 (5.7%) | 8 (4.8%) |
| *P. aeruginosa* | 9 (1.7%) | 15 (2.2%) | 14 (1.8%) | 21 (2.9%) | 28 (4.0%) | 7 (4.2%) |
| *Acinetobacter* spp. | 1 (0.2%) | 0 (0.0%) | 0 (0.0%) | 0 (0.0%) | 1 (0.1%) | 1 (0.6%) |
| 3GC-nonS Enterobacterales (excluding ECK) | 7 (1.3%) | 12 (1.8%) | 20 (2.6%) | 17 (2.4%) | 15 (2.1%) | 4 (2.4%) |
| ^1^Median (IQR); n (%) |  |  |  |  |  |  |

ECK: *Enterobacter cloacae* complex, *Citrobacter freundii*, *Klebsiella aerogenes*

3GC-nonS, third-generation cephalosporin-non-susceptible

Data were generated via the Antimicrobial and Patient Background Surveillance System (APBSS) using cases in which antimicrobial treatment was initiated by March 2024.

Table S4. Annual trends in the characteristics of patients with urinary tract infection at Toyota Kosei Hospital

| **Characteristics** | **2019**,  N = 248^1^ | **2020**,  N = 387^1^ | **2021**,  N = 400^1^ | **2022**,  N = 411^1^ | **2023**,  N = 411^1^ | **2024**,  N = 96^1^ |
| --- | --- | --- | --- | --- | --- | --- |
| Age, years | 78 (59, 86) | 78 (66, 86) | 79 (66, 87) | 78 (68, 86) | 78 (69, 87) | 82 (71, 87) |
| Sex |  |  |  |  |  |  |
| Female | 147 (59.3%) | 217 (56.1%) | 223 (55.8%) | 247 (60.1%) | 242 (58.9%) | 63 (65.6%) |
| Male | 101 (40.7%) | 170 (43.9%) | 177 (44.3%) | 164 (39.9%) | 169 (41.1%) | 33 (34.4%) |
| Length of therapy, days | 11 (7, 14) | 12 (8, 15) | 12 (8, 15) | 11 (8, 14) | 12 (8, 14) | 11 (7, 15) |
| Length of stay, days | 11 (7, 19) | 12 (8, 21) | 13 (8, 22) | 12 (7, 24) | 12 (8, 22) | 13 (8, 20) |
| Hospital mortality | 14 (5.7%) | 27 (7.0%) | 23 (5.8%) | 35 (8.5%) | 41 (10.0%) | 5 (5.2%) |
| Solid tumor | 38 (15.3%) | 53 (13.7%) | 63 (15.8%) | 63 (15.3%) | 75 (18.2%) | 7 (7.3%) |
| Hematological malignancy | 2 (0.8%) | 4 (1.0%) | 3 (0.8%) | 3 (0.7%) | 6 (1.5%) | 1 (1.0%) |
| Charlson Comorbidity Index | 1 (0, 2) | 1 (0, 2) | 1 (0, 2) | 1 (0, 2) | 2 (0, 2) | 0 (0, 2) |
| Detected bacteria |  |  |  |  |  |  |
| Enterobacterales ECK | 8 (3.2%) | 13 (3.4%) | 12 (3.0%) | 15 (3.6%) | 15 (3.6%) | 2 (2.1%) |
| *P. aeruginosa* | 23 (9.3%) | 23 (5.9%) | 25 (6.3%) | 25 (6.1%) | 33 (8.0%) | 9 (9.4%) |
| *Acinetobacter* spp. | 2 (0.8%) | 2 (0.5%) | 0 (0.0%) | 2 (0.5%) | 1 (0.2%) | 0 (0.0%) |
| 3GC-nonS Enterobacterales (excluding ECK) | 20 (8.1%) | 45 (11.6%) | 55 (13.8%) | 58 (14.1%) | 44 (10.7%) | 7 (7.3%) |
| ^1^Median (IQR); n (%) |  |  |  |  |  |  |

ECK: *Enterobacter cloacae* complex, *Citrobacter freundii*, *Klebsiella aerogenes*

3GC-nonS, third-generation cephalosporin-non-susceptible

Data were generated via the Antimicrobial and Patient Background Surveillance System (APBSS) using cases in which antimicrobial treatment was initiated by March 2024.

Table S5. Annual trends in the characteristics of patients with gram-negative rod bacteremia at Toyota Kosei Hospital

| **Characteristics** | **2019**,  N = 260^1^ | **2020**,  N = 371^1^ | **2021**,  N = 419^1^ | **2022**,  N = 372^1^ | **2023**,  N = 392^1^ | **2024**,  N = 96^1^ |
| --- | --- | --- | --- | --- | --- | --- |
| Age, years | 78 (70, 84) | 78 (70, 85) | 80 (72, 86) | 81 (72, 87) | 80 (72, 86) | 81 (74, 85) |
| Sex |  |  |  |  |  |  |
| Female | 115 (44.2%) | 176 (47.4%) | 169 (40.3%) | 178 (47.8%) | 181 (46.2%) | 47 (49.0%) |
| Male | 145 (55.8%) | 195 (52.6%) | 250 (59.7%) | 194 (52.2%) | 211 (53.8%) | 49 (51.0%) |
| Indication |  |  |  |  |  |  |
| CAI | 207 (79.6%) | 299 (80.6%) | 321 (76.6%) | 298 (80.1%) | 306 (78.1%) | 80 (83.3%) |
| HAI | 53 (20.4%) | 72 (19.4%) | 98 (23.4%) | 74 (19.9%) | 86 (21.9%) | 16 (16.7%) |
| Length of therapy, days | 13 (10, 15) | 13 (10, 17) | 13 (11, 17) | 13 (10, 17) | 13 (10, 16) | 13 (10, 15) |
| Length of stay, days | 15 (9, 29) | 15 (9, 30) | 17 (11, 36) | 15 (10, 29) | 16 (9, 32) | 16 (11, 27) |
| Hospital mortality | 30 (11.8%) | 37 (10.1%) | 61 (14.7%) | 44 (12.0%) | 51 (13.5%) | 14 (15.2%) |
| Solid tumor | 84 (32.3%) | 118 (31.8%) | 130 (31.0%) | 84 (22.6%) | 110 (28.1%) | 27 (28.1%) |
| Hematological malignancy | 4 (1.5%) | 6 (1.6%) | 12 (2.9%) | 4 (1.1%) | 6 (1.5%) | 1 (1.0%) |
| Charlson Comorbidity Index | 2 (0, 2) | 2 (0, 3) | 2 (0, 3) | 1 (0, 2) | 2 (0, 2) | 2 (0, 2) |
| Detected bacteria |  |  |  |  |  |  |
| Enterobacterales ECK | 17 (6.5%) | 29 (7.8%) | 52 (12.4%) | 26 (7.0%) | 30 (7.7%) | 12 (12.5%) |
| *P. aeruginosa* | 16 (6.2%) | 21 (5.7%) | 26 (6.2%) | 23 (6.2%) | 26 (6.6%) | 6 (6.3%) |
| *Acinetobacter* spp. | 4 (1.5%) | 2 (0.5%) | 4 (1.0%) | 0 (0.0%) | 3 (0.8%) | 1 (1.0%) |
| 3GC-nonS Enterobacterales (excluding ECK) | 34 (13.1%) | 42 (11.3%) | 64 (15.3%) | 42 (11.3%) | 50 (12.8%) | 8 (8.3%) |
| ^1^Median (IQR); n (%) |  |  |  |  |  |  |

CAIs: community-acquired infection

HAIs: healthcare-associated infections.

ECK: *Enterobacter cloacae* complex, *Citrobacter freundii*, *Klebsiella aerogenes*

3GC-nonS, third-generation cephalosporin-non-susceptible

Data were generated via the Antimicrobial and Patient Background Surveillance System (APBSS) using cases in which antimicrobial treatment was initiated by March 2024.

Table S6. Characteristics of patients with pneumonia in various hospitals

| **Characteristics** | **Hospital A**,  N = 4,755^1^ | **Hospital B**,  N = 4,086^1^ | **Hospital C**,  N = 5,056^1^ | **Toyota Kosei Hospital**,  N = 4,911^1^ |
| --- | --- | --- | --- | --- |
| Age, years | 77 (60, 85) | 81 (73, 87) | 80 (67, 87) | 81 (71, 87) |
| Sex |  |  |  |  |
| Female | 1,797 (37.8%) | 1,457 (35.7%) | 1,935 (38.3%) | 1,850 (37.7%) |
| Male | 2,958 (62.2%) | 2,629 (64.3%) | 3,121 (61.7%) | 3,061 (62.3%) |
| ADROP score |  |  |  |  |
| 0 | 119 (2.5%) | 155 (3.8%) | 192 (3.8%) | 179 (3.6%) |
| 1 or 2 | 1,082 (22.8%) | 1,116 (27.3%) | 1,538 (30.4%) | 1,205 (24.5%) |
| 3 | 489 (10.3%) | 413 (10.1%) | 559 (11.1%) | 506 (10.3%) |
| 4 or 5 | 251 (5.3%) | 109 (2.7%) | 207 (4.1%) | 200 (4.1%) |
| Unknown | 2,814 (59.2%) | 2,293 (56.1%) | 2,560 (50.6%) | 2,821 (57.4%) |
| Length of therapy, days | 8 (6, 13) | 10 (7, 15) | 9 (7, 14) | 10 (7, 14) |
| Length of stay, days | 14 (7, 26) | 16 (9, 29) | 15 (8, 31) | 17 (9, 30) |
| Hospital mortality | 815 (17.2%) | 725 (17.9%) | 907 (18.2%) | 901 (18.6%) |
| Solid tumor | 719 (15.1%) | 505 (12.4%) | 538 (10.6%) | 804 (16.4%) |
| Hematological malignancy | 152 (3.2%) | 96 (2.3%) | 132 (2.6%) | 126 (2.6%) |
| Charlson Comorbidity Index | 0 (0, 2) | 1 (0, 2) | 1 (0, 2) | 2 (0, 3) |
| Detected bacteria |  |  |  |  |
| *S. pneumoniae* | 145 (3.0%) | 128 (3.1%) | 128 (2.5%) | 166 (3.4%) |
| *M. catarrhalis* | 80 (1.7%) | 59 (1.4%) | 77 (1.5%) | 123 (2.5%) |
| *H. influenzae* | 174 (3.7%) | 104 (2.5%) | 170 (3.4%) | 218 (4.4%) |
| Enterobacterales ECK | 324 (6.8%) | 230 (5.6%) | 134 (2.7%) | 251 (5.1%) |
| *P. aeruginosa* | 394 (8.3%) | 250 (6.1%) | 242 (4.8%) | 342 (7.0%) |
| *Acinetobacter* spp. | 73 (1.5%) | 73 (1.8%) | 16 (0.3%) | 72 (1.5%) |
| 3GC-nonS Enterobacterales (excluding ECK) | 152 (3.2%) | 266 (6.5%) | 118 (2.3%) | 150 (3.1%) |
| ^1^Median (IQR); n (%) |  |  |  |  |

ECK: *Enterobacter cloacae* complex, *Citrobacter freundii*, *Klebsiella aerogenes*

3GC-nonS, third-generation cephalosporin-non-susceptible

Data were generated via the Antimicrobial and Patient Background Surveillance System (APBSS) using cases in which antimicrobial treatment was initiated by March 2024.

Table S7. Characteristics of patients with intra-abdominal infections at various hospitals

| **Characteristics** | **Hospital A**,  N = 3,474^1^ | **Hospital B**,  N = 2,611^1^ | **Hospital C**,  N = 2,729^1^ | **Toyota Kosei Hospital**,  N = 3,559^1^ |
| --- | --- | --- | --- | --- |
| Age, years | 72 (57, 81) | 74 (61, 82) | 74 (58, 82) | 73 (58, 83) |
| Sex |  |  |  |  |
| Female | 1,356 (39.0%) | 1,131 (43.3%) | 1,203 (44.1%) | 1,461 (41.1%) |
| Male | 2,118 (61.0%) | 1,480 (56.7%) | 1,526 (55.9%) | 2,098 (58.9%) |
| Length of therapy, days | 9 (7, 14) | 9 (6, 13) | 9 (6, 13) | 9 (7, 14) |
| Length of stay, days | 11 (8, 18) | 11 (7, 18) | 11 (8, 19) | 11 (7, 19) |
| Hospital mortality | 200 (5.8%) | 138 (5.3%) | 171 (6.4%) | 171 (4.8%) |
| Solid tumor | 852 (24.5%) | 680 (26.0%) | 686 (25.1%) | 852 (23.9%) |
| Hematological malignancy | 29 (0.8%) | 25 (1.0%) | 10 (0.4%) | 26 (0.7%) |
| Charlson Comorbidity Index | 0 (0, 2) | 0 (0, 2) | 0 (0, 2) | 0 (0, 2) |
| Detected bacteria |  |  |  |  |
| Enterobacterales ECK | 180 (5.2%) | 106 (4.1%) | 132 (4.8%) | 187 (5.3%) |
| *P. aeruginosa* | 44 (1.3%) | 30 (1.1%) | 48 (1.8%) | 94 (2.6%) |
| *Acinetobacter* spp. | 2 (0.1%) | 5 (0.2%) | 4 (0.1%) | 3 (0.1%) |
| 3GC-nonS Enterobacterales (excluding ECK) | 113 (3.3%) | 87 (3.3%) | 94 (3.4%) | 75 (2.1%) |
| ^1^Median (IQR); n (%) |  |  |  |  |

ECK: *Enterobacter cloacae* complex, *Citrobacter freundii*, *Klebsiella aerogenes*

3GC-nonS, third-generation cephalosporin-non-susceptible

Data were generated via the Antimicrobial and Patient Background Surveillance System (APBSS) using cases in which antimicrobial treatment was initiated by March 2024.

Table S8. Characteristics of patients with urinary tract infection at various hospitals

| **Characteristics** | **Hospital A**,  N = 1,522^1^ | **Hospital B**,  N = 1,873^1^ | **Hospital C**,  N = 1,792^1^ | **Toyota Kosei Hospital**,  N = 1,953^1^ |
| --- | --- | --- | --- | --- |
| Age, years | 74 (42, 84) | 80 (71, 87) | 81 (70, 87) | 78 (67, 87) |
| Sex |  |  |  |  |
| Female | 763 (50.1%) | 1,018 (54.4%) | 1,071 (59.8%) | 1,139 (58.3%) |
| Male | 759 (49.9%) | 855 (45.6%) | 721 (40.2%) | 814 (41.7%) |
| Length of therapy, days | 12 (8, 15) | 12 (8, 15) | 11 (8, 14) | 12 (8, 14) |
| Length of stay, days | 10 (7, 18) | 13 (8, 21) | 14 (9, 28) | 12 (7, 21) |
| Hospital mortality | 88 (5.8%) | 129 (6.9%) | 190 (10.8%) | 145 (7.5%) |
| Solid tumor | 198 (13.0%) | 224 (12.0%) | 217 (12.1%) | 299 (15.3%) |
| Hematological malignancy | 25 (1.6%) | 20 (1.1%) | 22 (1.2%) | 19 (1.0%) |
| Charlson Comorbidity Index | 0 (0, 1) | 0 (0, 2) | 0 (0, 2) | 1 (0, 2) |
| Detected bacteria |  |  |  |  |
| Enterobacterales ECK | 62 (4.1%) | 55 (2.9%) | 63 (3.5%) | 65 (3.3%) |
| *P. aeruginosa* | 86 (5.7%) | 97 (5.2%) | 80 (4.5%) | 138 (7.1%) |
| *Acinetobacter* spp. | 6 (0.4%) | 9 (0.5%) | 7 (0.4%) | 7 (0.4%) |
| 3GC-nonS Enterobacterales (excluding ECK) | 184 (12.1%) | 317 (16.9%) | 219 (12.2%) | 229 (11.7%) |
| ^1^Median (IQR); n (%) |  |  |  |  |

ECK: *Enterobacter cloacae* complex, *Citrobacter freundii*, *Klebsiella aerogenes*

3GC-nonS, third-generation cephalosporin-non-susceptible

Data were generated via the Antimicrobial and Patient Background Surveillance System (APBSS) using cases in which antimicrobial treatment was initiated by March 2024.

Table S9. Characteristics of patients with gram-negative rod bacteremia at various hospitals

| **Characteristics** | **Hospital A**,  N = 1,927^1^ | **Hospital B**,  N = 1,533^1^ | **Hospital C**,  N = 1,467^1^ | **Toyota Kosei Hospital**,  N = 1,910^1^ |
| --- | --- | --- | --- | --- |
| Age, years | 77 (69, 84) | 80 (73, 86) | 80 (72, 86) | 79 (72, 86) |
| Sex |  |  |  |  |
| Female | 848 (44.0%) | 689 (44.9%) | 730 (49.8%) | 866 (45.3%) |
| Male | 1,079 (56.0%) | 844 (55.1%) | 737 (50.2%) | 1,044 (54.7%) |
| Indication |  |  |  |  |
| CAI | 1,531 (79.4%) | 1,224 (79.8%) | 1,129 (77.0%) | 1,511 (79.1%) |
| HAI | 396 (20.6%) | 309 (20.2%) | 338 (23.0%) | 399 (20.9%) |
| Length of therapy, days | 14 (10, 18) | 14 (10, 17) | 13 (10, 16) | 13 (10, 16) |
| Length of stay, days | 16 (9, 31) | 16 (10, 32) | 18 (10, 37) | 16 (10, 31) |
| Hospital mortality | 344 (18.1%) | 206 (13.6%) | 242 (16.9%) | 237 (12.7%) |
| Solid tumor | 539 (28.0%) | 349 (22.8%) | 325 (22.2%) | 553 (29.0%) |
| Hematological malignancy | 159 (8.3%) | 58 (3.8%) | 74 (5.0%) | 33 (1.7%) |
| Charlson Comorbidity Index | 1 (0, 2) | 1 (0, 2) | 2 (0, 2) | 2 (0, 2) |
| Detected bacteria |  |  |  |  |
| Enterobacterales ECK | 181 (9.4%) | 101 (6.6%) | 114 (7.8%) | 166 (8.7%) |
| *P. aeruginosa* | 87 (4.5%) | 82 (5.3%) | 89 (6.1%) | 118 (6.2%) |
| *Acinetobacter* spp. | 11 (0.6%) | 14 (0.9%) | 17 (1.2%) | 14 (0.7%) |
| 3GC-nonS Enterobacterales (excluding ECK) | 319 (16.6%) | 333 (21.7%) | 216 (14.7%) | 240 (12.6%) |
| ^1^Median (IQR); n (%) |  |  |  |  |

CAIs: community-acquired infections

HAIs: healthcare-associated infections.

ECK: *Enterobacter cloacae* complex, *Citrobacter freundii*, *Klebsiella aerogenes*

3GC-nonS, third-generation cephalosporin-non-susceptible

Data were generated via the Antimicrobial and Patient Background Surveillance System (APBSS) using cases in which antimicrobial treatment was initiated by March 2024.

Table S10 Parameter estimates from the interrupted time series analysis model of Days of therapy for carbapenem

|  | **Estimate** | **95% CI**^1^ | **p-value** |
| --- | --- | --- | --- |
| (Intercept) | 2.9 | 2.6, 3.2 | <0.001 |
| Pre slope | -0.02 | -0.04, 0.01 | 0.13 |
| Level change | -0.69 | -1.2, -0.13 | 0.017 |
| Slope change | 0.07 | 0.00, 0.13 | 0.045 |
| ^1^CI = Confidence Interval | | | |

Table S11 Parameter estimates from the interrupted time series analysis model of Days of therapy for carbapenem excluding use in the hematology department

|  | **Estimate** | **95% CI**^1^ | **p-value** |
| --- | --- | --- | --- |
| (Intercept) | 2.3 | 2.1, 2.6 | <0.001 |
| Pre slope | -0.01 | -0.03, 0.01 | 0.40 |
| Level change | -0.60 | -1.1, -0.15 | 0.011 |
| Slope change | 0.04 | -0.02, 0.09 | 0.20 |
| ^1^CI = Confidence Interval | | | |

Table S12 Parameter estimates from the interrupted time series analysis model of Days of therapy for antipseudomonal agents

|  | **Estimate** | **95% CI**^1^ | **p-value** |
| --- | --- | --- | --- |
| (Intercept) | 5.5 | 5.0, 6.1 | <0.001 |
| Pre slope | 0.10 | 0.05, 0.14 | <0.001 |
| Level change | -1.7 | -2.8, -0.58 | 0.004 |
| Slope change | 0.03 | -0.10, 0.16 | 0.70 |
| ^1^CI = Confidence Interval | | | |

Table S13 Parameter estimates from the interrupted time series analysis model of Days of therapy for carbapenem in hospitals with additional healthcare reimbursement for infection prevention and control fee 1

|  | **Estimate** | **95% CI**^1^ | **p-value** |
| --- | --- | --- | --- |
| (Intercept) | 2.0 | 1.9, 2.0 | <0.001 |
| Pre slope | -0.01 | -0.02, -0.01 | <0.001 |
| Level change | 0.08 | -0.03, 0.19 | 0.13 |
| Slope change | 0.00 | -0.02, 0.01 | 0.70 |
| ^1^CI = Confidence Interval | | | |

Table S14 Parameter estimates from the interrupted time series analysis model of Days of therapy for carbapenem in four infections

|  | **Estimate** | **95% CI**^1^ | **p-value** |
| --- | --- | --- | --- |
| Pneumonia |  |  |  |
| (Intercept) | 0.65 | 0.51, 0.79 | <0.001 |
| Pre slope | -0.01 | -0.02, 0.00 | 0.20 |
| Level change | -0.03 | -0.31, 0.25 | 0.80 |
| Slope change | 0.01 | -0.02, 0.04 | 0.60 |
| Intra-abdominal infection |  |  |  |
| (Intercept) | 0.49 | 0.37, 0.62 | <0.001 |
| Pre slope | 0.00 | -0.01, 0.01 | 0.80 |
| Level change | -0.23 | -0.48, 0.02 | 0.068 |
| Slope change | 0.01 | -0.02, 0.04 | 0.60 |
| Pyelonephritis |  |  |  |
| (Intercept) | 0.07 | 0.01, 0.14 | 0.032 |
| Pre slope | 0.01 | 0.00, 0.01 | 0.007 |
| Level change | -0.14 | -0.26, -0.01 | 0.038 |
| Slope change | -0.01 | -0.03, 0.00 | 0.090 |
| Gram-negative rod bacteremia |  |  |  |
| (Intercept) | 0.28 | 0.14, 0.42 | <0.001 |
| Pre slope | 0.01 | 0.00, 0.02 | 0.013 |
| Level change | -0.30 | -0.57, -0.04 | 0.027 |
| Slope change | 0.00 | -0.04, 0.03 | 0.70 |
| ^1^CI = Confidence Interval | | | |
